# Colonization of the deep sea by fishes

**DOI:** 10.1111/jfb.12265

**Published:** 2013-12-03

**Authors:** I G Priede, R Froese

**Affiliations:** * Oceanlab, Institute of Biological and Environmental Sciences, University of AberdeenMain Street, Newburgh, Aberdeen AB41 6AA, U.K.; ‡ GEOMAR Helmholtz-Centre for Ocean ResearchDuesternbrooker Weg 20, Kiel 24105, Germany

**Keywords:** Actinopterygii, Chondrichthyes, Cretaceous, evolution, global anoxic event, Myxini

## Abstract

Analysis of maximum depth of occurrence of 11 952 marine fish species shows a global decrease in species number (*N*) with depth (*x*; m): log_10_*N* = −0·000422*x* + 3·610000 (*r*^2^ = 0·948). The rate of decrease is close to global estimates for change in pelagic and benthic biomass with depth (−0·000430), indicating that species richness of fishes may be limited by food energy availability in the deep sea. The slopes for the Classes Myxini (−0·000488) and Actinopterygii (−0·000413) follow this trend but Chondrichthyes decrease more rapidly (−0·000731) implying deficiency in ability to colonize the deep sea. Maximum depths attained are 2743, 4156 and 8370 m for Myxini, Chondrichthyes and Actinopterygii, respectively. Endemic species occur in abundance at 7–7800 m depth in hadal trenches but appear to be absent from the deepest parts of the oceans, >9000 m deep. There have been six global oceanic anoxic events (OAE) since the origin of the major fish taxa in the Devonian *c*. 400 million years ago (mya). Colonization of the deep sea has taken place largely since the most recent OAE in the Cretaceous 94 mya when the Atlantic Ocean opened up. Patterns of global oceanic circulation oxygenating the deep ocean basins became established coinciding with a period of teleost diversification and appearance of the Acanthopterygii. Within the Actinopterygii, there is a trend for greater invasion of the deep sea by the lower taxa in accordance with the Andriashev paradigm. Here, 31 deep-sea families of Actinopterygii were identified with mean maximum depth >1000 m and with >10 species. Those with most of their constituent species living shallower than 1000 m are proposed as invasive, with extinctions in the deep being continuously balanced by export of species from shallow seas. Specialized families with most species deeper than 1000 m are termed deep-sea endemics in this study; these appear to persist in the deep by virtue of global distribution enabling recovery from regional extinctions. Deep-sea invasive families such as Ophidiidae and Liparidae make the greatest contribution to fish fauna at depths >6000 m.

## INTRODUCTION

Recent research on the origins of life on Earth increasingly favours the deep sea as the most likely site of the transition from geochemistry to life, with alkaline hydrothermal vents providing optimal conditions for this step (Lane & Martin, 2012). This is in contrast to the azoic hypothesis of Forbes (1844) who proposed that at depths >550 m the oceans are devoid of life. Despite this assertion, it is likely that from occasional accidental catches, strandings and carcasses floating to the surface, humans have long been aware that the oceans may harbour a deep-sea fish fauna. Working in the Mediterranean Sea, Risso (1810) provided one of the first scientific accounts of deep-sea fish species. He describes common mora *Gadus moro*, now known as *Mora moro* (Risso 1810), as very common at great depths in the Sea of Nice with very tender white flesh and good flavour. He observed depth zonation with species including chimaeras and Risso's smooth-head *Alepocephalus rostratus* Risso 1820 occurring at the greatest depths and lings, whitings and Phycidae at shallower depths (Risso, 1826). His descriptions are reiterated in the English language edition of the Universal Geography of Malte-Brun (1827). In 1833, Lowe began publishing a series of papers with descriptions of fishes found around the island of Madeira in the north-east Atlantic Ocean including black scabbard fish *Aphanopus carbo* Lowe 1839 and gives an account of how fishermen retrieve wreckfish *Polyprion americanus* (Bloch & Schneider 1801) from 550 to 730 m depth using baited lines (Lowe, 1843–1860). It is therefore surprising that some years later Forbes (1844) proposed the azoic hypothesis but this was based on the analysis of samples of benthic invertebrates dredged from the Aegean Sea. Anderson & Rice (2006) point out that there was substantial evidence before 1844 of invertebrate life at great depths in the oceans that appears to have been simply ignored by Forbes and some of his contemporaries.

There is no doubt that major advances in understanding of deep-sea fishes were made by Günther (1887) through the study of samples collected during the circumnavigation of the globe by the HMS *Challenger* (1873–1876) and associated expeditions that sampled depths to 2900 fathoms (ftm; 5300 m). The *Challenger* expedition finally refuted the azoic hypothesis demonstrating that life occurs even at great depths in the oceans. A total of 385 fish species were found at depths >100 ftm (183 m) but Günther only recognized 230 species found at depths >550 m as truly deep-sea species. Numbers of species and individuals were found to decrease with depth; Macrouridae being the most abundant followed by Ophidiidae and Gadidae. In his chapter on deep-sea fishes, Günther (1880) wrote ‘*the fish fauna of the deep sea is composed chiefly of forms or modifications of forms we find represented at the surface*…’. He concluded that 2750 ftm (5030 m) must be accepted as a depth at which fishes undoubtedly do live but the deepest species referred to in his work were almost certainly caught at shallower depths during ascent of the dredge to the surface. Since that time, cumulative sampling effort has demonstrated that fishes occur throughout the abyssal regions of the world's oceans down to over 6000 m depth (Merrett & Haedrich, 1997). In the 1950s, the expeditions by the *Galathea* (1950–1952) from Denmark and the *Vityaz* (1953–1957) from Russia undertook systematic sampling at depths >6000 m in hadal trenches of the Pacific Ocean. They found liparids, *Notoliparis kermadecensis* (Nielsen 1964) at 6660–6770 m depth in the Kermadec Trench in the south-west Pacific Ocean and *Pseudoliparis amblystomopsis* (Andriashev 1955) at 7230–7579 m in the Kuril-Kamchatka and Japan trenches of the north-west Pacific Ocean (Nielsen, 1964; Fujii *et al*., 2010). The deepest fish recorded to date is *Abyssobrotula galatheae* Nielsen 1977, retrieved in 1970 from 8370 m depth in the Puerto Rico Trench. By the first decade of the 21st century, 3356 species of deep-sea fishes had been described, comprising 2081 bathydemersal and 1275 bathypelagic species. Extrapolating trends of species discovery, Mora *et al*. (2008) estimated that 1638 bathydemersal and 395 bathypelagic species remain to be discovered giving an expected total deep-sea ichthyofauna of 5389 species.

Woodward (1898) considered the origin of deep-sea fishes and argued that the evolutionary struggle for existence is most intense along the shore-line and that fresh waters and deep sea can be regarded as refuges into which less competitive primitive taxa have retreated. In Cretaceous chalk deposits, he identified fossils very similar to modern deep-sea fishes such as *Sardinius* now assigned to the Myctophiformes (Sepkoski, 2002) and *Echidnocephalus*, a halosaur. Thus, morphologically distinguishable deep water types evolved quite soon after the appearance of the first teleosts in the fossil record. He concludes that colonization of the deep sea by fishes has been a gradual process from Cretaceous times to the present day with a succession of new taxa moving deeper from the shallow seas in which they first arose. Andriashev (1953) divided the deep-sea fish fauna into ancient (or true) deep-water forms and secondary deep-water forms. The former correspond to the early deep-sea fishes belonging to lower phylogenetic groups such as Clupeiformes, Anguiliformes and Gadiformes and are characterized by world-wide distribution, specialized morphology and occupation of the greatest depths in the oceans. He includes Holocephali and Selachii in the ancient group. The secondary deep-water forms belong to the more recent teleost taxa, mainly Perciformes and the rays. His thinking was influenced by studies of the Arctic Ocean. He noted that relatively warm waters of the Atlantic Ocean, south of the Wyville Thompson Ridge, are populated by the ancient deep-water ichthyofauna, whereas these species are absent in cold waters to the north where secondary deep-water species derived from adjacent shelf areas are prevalent.

Thus, although life may have originated in the deep sea, colonization of the deep sea by fishes appears to have been through a process of diversification from shallow waters. Present day patterns of distribution of fishes are likely to reflect this history. Priede *et al*. (2006) pointed out that the Class Chondrichthyes is largely absent from abyssal depths >3000 m, whereas members of the Class Actinopterygii are found down to over 8000 m. In this paper, the depth distribution patterns of the modern fishes are examined and these are interpreted in relation to the evolution of deep-sea fishes.

## MATERIALS AND METHODS

Data for depths of occurrence and maximum lengths were obtained for all marine species listed in fishbase.org (Froese & Pauly, 2013). This yielded entries for 16 307 species with records of maximum depths for 11 952 species. Depth data tend to be missing for shallow-water species for which depth is not regarded as a significant environmental variable. Data were sorted taxonomically and Petromyzontiformes (the few marine representatives are all anadromous), Pristiformes (shallow-water estuarine and freshwater habitats), Coelocanthiformes (Class Sarcopterygii, two living species, cave-dwelling, maximum depth 700 m) and Cyprinodontiformes (freshwater order with a few shallow-water marine representatives) were excluded from the analysis. The analysis was based on the maximum depth recorded for each species which is not representative of the typical depth at which most individuals of a species are likely to be found. Maximum depth, however, has the advantage that it has been recorded for most species and is an unequivocal single number. Caution is required when fishes have been captured using non-closing tow nets as specimens may have entered the net at any stage during its ascent often from great depths. Maximum depths over 6000 m were checked against the review by Fujii *et al*. (2010). Records were considered suspect if the depth was much deeper than typical for the species or group of species and where depth recorded was the maximum reached by gear that traversed a wide depth range and the specimen could have been captured at much shallower depths as in the case of a non-closing net. Some instances in which the depth recorded was the bottom sounding over which a pelagic net was towed were corrected to the actual depth of the net. For analysis of depth distributions, data were clustered in 500 m depth strata, 0–499, 500–999, 1000–1499 m *et seq*.

## RESULTS

### COMPARISON OF CLASSES MYXINI, CHONDRICHTHYES AND ACTINOPTERYGII

**Fig 1:**
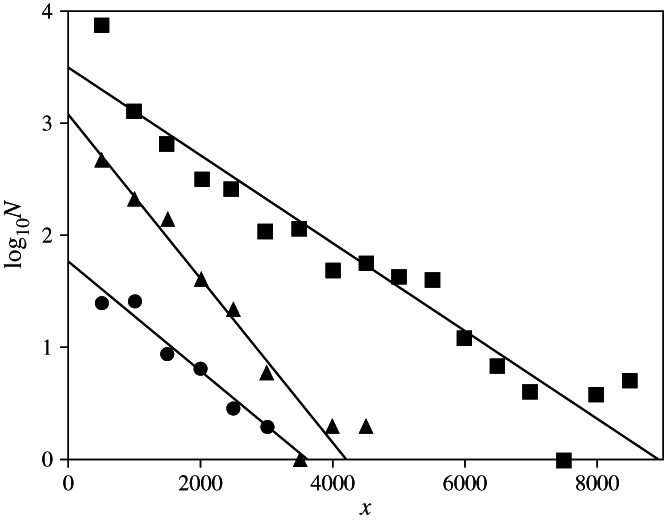
The relationship between log_10_*N* (*N* = number of species within a 500 m depth stratum) and depth (*x*) for three classes of fishes with fitted regression lines: Myxini (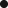) log_10_*N* = −0·000488*x* + 1·760000 (*r*^2^ = 0·965), Chondricthyes (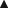) log_10_*N* = −0·000731*x* + 3·070000 (*r*^2^ = 0·954) and Actinopterygii (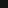) log_10_*N* = −0·000413*x* + 3·550000 (*r*^2^ = 0·944).

There was an overall trend of logarithmic decrease in species number with depth described by the fitted regression equation: all marine fish species log_10_*N* = −0·000422*x* + 3·610000 (*r*^2^ = 0·948), where *N* is the number of species maximum depths per 500 m depth stratum and *x* is the depth (m). Similar trends were seen for the individual classes (Fig. 1).

**Fig 2:**
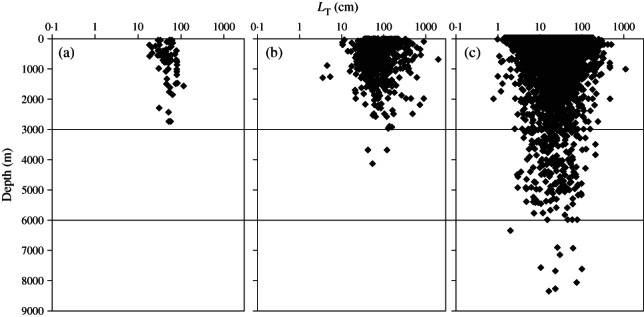
Scatter plots of species' maximum depth of occurrence and maximum total length (*L*_T_) for three classes of fishes: (a) Myxini, (b) Chondrichthyes and (c) Actinopterygii.

The global database contains records for 78 species of Myxini with data for 74 of these with a mean ± s.d. maximum depth of 795 ± 647 m. Two species of hagfishes were recorded at the maximum depth for this class with 2743 m, black hagfish *Eptatretus deani* (Evermann & Goldsborough 1907) and Guadalupe hagfish *Eptatretus fritzi* Wisner & McMillan 1990 (Fig. 2 and Table1). Number of species decreased with depth from 26 species found at shallower depths than 500 m, 27 species at 500–999 m and two species found deeper than 2500 m. The mean ± s.d. maximum total length (*L*_T_) was 51·2 ±18·5 cm with no obvious change in size with depth (Fig. 2).

**Table 1:** List of orders of fishes with statistics for maximum depths of occurrence. Orders are numbered in sequence of appearance in FishBase (Froese & Pauly, 2013). Statistics are given for the maximum depths

						Maximum depths (m)			
Class	Andriashev (1953)		Order		*n*	Mean	s.d.	Minimum	Maximum
Myxni	Ancient	Myxini	1	Myxiniformes	74	795	647	40	2743
Chondrichthyes		Holocephali	2	Chimaeriformes	45	1168	665	116	3000
		Selachii	3	Hexanchiformes	6	1273	725	570	2500
			4	Heterodontiformes	9	132	97	37	275
			5	Orectolobiformes	33	109	127	3	700
			6	Lamniformes	15	856	568	191	2000
			7	Carcharhiniformes	224	560	531	4	4000
			8	Squaliformes	124	985	622	100	3700
			9	Pristiophoriformes	7	473	260	165	1000
			10	Squatiniformes	18	368	286	100	1390
	Secondary	Batoidea	11	Torpediniformes	46	310	291	17	1153
			12	Rajiformes	385	598	612	6	4156
Actinopterygii	Ancient	Lower Teleosts	13	Elopiformes	1	50		50	50
			14	Albuliformes	4	419	434	84	1000
			15	Notacanthiformes	27	2045	1112	706	5029
			16	Anguilliformes	590	463	805	1	6000
			17	Saccopharyngiformes	23	2765	1635	350	5800
			18	Clupeiformes	163	67	71	2	450
			19	Gonorynchiformes	5	375	481	100	1233
			20	Siluriformes	20	45	29	5	120
			21	Osmeriformes	203	1654	1139	70	5850
			22	Stomiiformes	329	1308	1197	50	5301
			23	Ateleopodiformes	10	675	234	494	1281
			24	Aulopiformes	208	1184	1399	6	5900
			25	Myctophiformes	210	1096	867	90	6000
			26	Lampriformes	14	558	359	90	1200
			27	Polymixiiformes	10	530	173	250	770
		Paracanthopterygii	28	Gadiformes	562	1139	937	10	6945
			29	Ophidiiformes	442	1139	1602	1	8370
			30	Batrachoidiformes	32	117	129	3	600
			31	Lophiiformes	297	1071	950	3	6370
	Secondary	Acanthopterygii	32	Gobiesociformes	58	49	77	0	348
			33	Atheriniformes	12	10	12	1	39
			34	Beloniformes	47	20	32	1	230
			35	Stephanoberyciformes	59	2043	1367	200	5397
			36	Beryciformes	140	389	582	3	4992
			37	Cetomimiformes	28	2285	1359	200	6200
			38	Zeiformes	32	827	444	200	1900
			39	Gasterosteiformes	6	108	93	30	291
			40	Syngnathiformes	190	67	125	1	1000
			41	Scorpaeniformes	1238	631	869	1	7703
			42	Perciformes	5206	200	460	0	5691
			43	Pleuronectiformes	542	280	365	1	3210
			44	Tetraodontiformes	253	152	235	5	2000
			45	Mugiliformes	3	10	9	4	20

For the Chondrichthyes, 1141 species records were available with maximum depth records for 912 species. The deepest species was Bigelow's ray *Rajella bigelowi* (Stehmann 1978) recorded at 4156 m. More than 50% of species (475 records) occurred at depths <500 m with a mean ± s.d. maximum depth of 636 ± 608 m. The range of *L*_T_ was greatest at shallower depths (Fig. 2) and overall mean ± s.d. maximum *L*_T_ was 108·5 ± 119·3 cm.

For the Actinopterygii, there were 15 085 species, with maximum depth data for 10 965 of these. A total of 72% of species were recorded at depths shallower than 500 m but the distribution extends to 8370 m. The record for *A. galatheae* is the deepest known occurrence (Fig. 2). The mean ± s.d. maximum depth was 497·7 ± 879·6 m. There was no trend of change of fish size with depth and mean ± s.d. maximum *L*_T_ was 33·4 ± 47·8 cm with the widest size range occurring at shallowest depths.

### THE CHONDRICHTHYES

#### Subclass Holocephali

**Fig 3:**
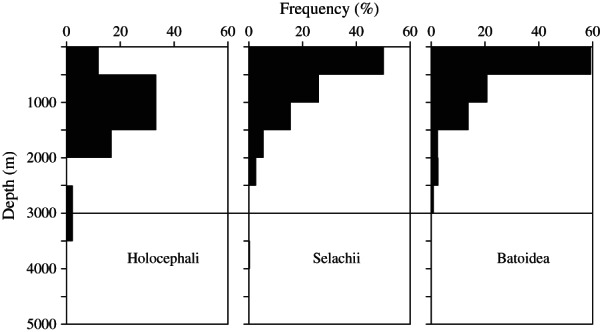
Chondrichthyes. The depth-frequency distributions of three taxa, subclass Holocephali, subdivision Selachii and subdivision Batoidea. The horizontal line at 3000 m depth delineates the upper boundary of the abyss from which Chondrichthyes are largely absent (Priede *et al.*, 2006).

The single order of Holocephali contained 50 species with maximum depth records for 45 species. Five species had maximum depths <500 m and 64% of species records are between 500 and 1500 m (Fig. 3). The mean ± s.d. maximum depth was 1168 ± 657 m and the deepest species was the smalleyed rabbitfish *Hydrolagus affinis* (de Brito Capello 1868) recorded at 3000 m depth.

#### Subclass Elasmobranchii, subdivision Selachii (sharks)

The Selachii comprises eight families (Table1) with a total of 504 species and 436 records of maximum depth of occurrence. Depths <500 m accounted for 49·9% of species and there was a significant decrease in species number with depth (Fig. 3): log_10_*N* = −0·000687*x* + 2·6700 (*r*^2^ = 0·880). The two deepest species were the cookie-cutter shark *Isistius brasiliensis* (Quoy & Gaimard 1824) and the Portuguese dogfish *Centroscymnus coelolepis* Barbosa du Bocage & de Brito Capello 1864, both recorded at 3700 m. The mean ± s.d. depth was 641 ± 569 m.

#### Subclass Elasmobranchii, subdivision Batoidea (rays)

The Batoidea comprises 587 species from two orders (Table1) with a total of 431 depth records. A total of 59·3% of species records were for depths <500 m and the mean ± s.d. maximum depth of occurrence was 568 ± 592 m. The deepest species in the order Torpediniformes was the New Zealand torpedo *Torpedo fairchildi* Hutton 1872 recorded at 1153 m and the deepest Rajiform species was *R. bigelowi* already referred to as the deepest of the Chondrichthyes at 4156 m. There was a significant decrease in species number with depth: log_10_*N* = −0·000605*x* + 2·550000 (*r*^2^ = 0·951).

### ACTINOPTERYGII

**Fig 4:**
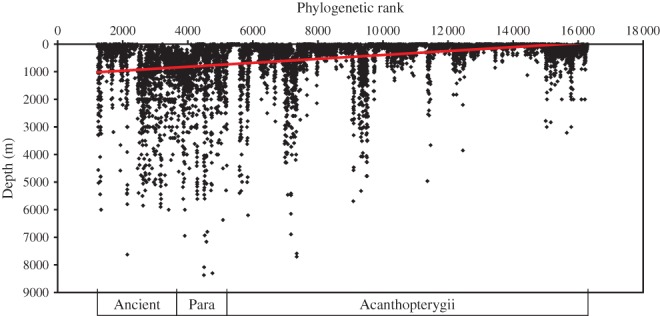
Scatterplot of Actinopterygii species' maximum depth of occurrence in relation to phylogenetic rank in FishBase with a linear trend line fitted to the data: *y* = −0·0711*x* + 1103·6000 (*r*^2^ = 0·125).

The Actinopterygii comprise 33 orders arranged in the phylogenetic sequence shown in Table1. The sequence from FishBase was used, which is based on a preliminary phylogeny, ranking species according to the presumed age of their common ancestor at least to their order level and within order and family wherever possible (Preikshot *et al*., 2000). Within each genus, species are ranked alphabetically. The relationship between maximum depth of occurrence and species rank is shown in Fig. 4. Whilst most species of Actinopterygii occur shallower than 500 m, the vertical linear clusters of points indicate taxa that have extended their range into the deep sea beyond 1000 m depth. There is a general trend that these and the maximum depths attained both decrease with increase in phylogenetic rank number. This is reflected in the linear fitted trend line indicating a decline in species depth: *y* = −0·0711*x* + 1104·0000 (*r*^2^ = 0·125), where *y* is depth in m and *x* is species taxonomic rank number. Although the correlation coefficient is low (*i.e*. there are other explanatory variables), the very large sample size (10 695) makes the effect of phylogeny highly significant (*P* < 0·001). It is interesting to note that the trend is also significant within the Acanthopterygii.

**Fig 5:**
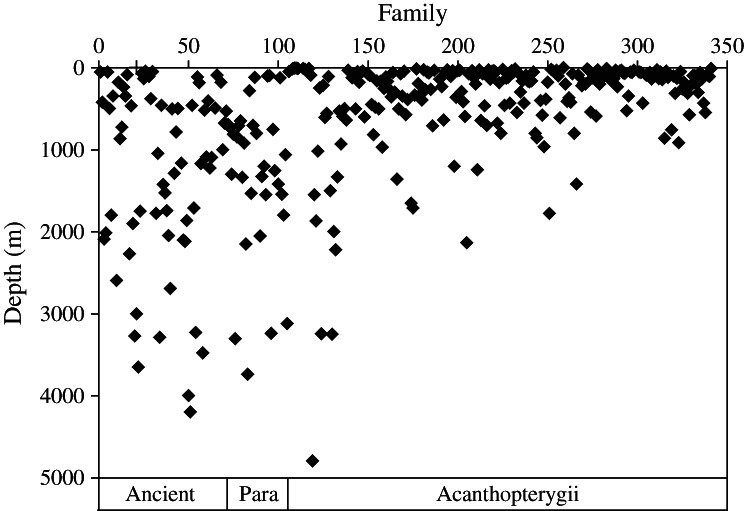
Mean maximum depth of occurrence by actinopterygian family in relation to phylogenetic rank according the FishBase.

Grouping the data into 341 families, the mean depth of occurrence by family shows the same trend of decrease in depth from the ancient to advanced taxa (Fig. 5). To identify the most important deep-sea families of Actinopterygii, all families with a mean maximum depth of occurrence >1000 m were selected. Of these, 31 families had 10 or more species and are listed in Table2. The ancient or lower teleost orders contribute 17 families, the Paracanthopterygii eight and the Acanthopterygii contribute six families. Lower teleost families, however, have fewer species so the contributions to the deep-sea ichthyofauna were more or less equal, with lower teleosts of 953 species, Paracanthopterygii 824 and Acanthopterygii of 803 species. Mean maximum depths were 1815, 1695 and 1693 m, respectively.

**Table 2:** List of deep-sea actinopterygian families with mean maximum depth >1000 m and with >10 species. Numbering (N) is in the order of listing in FishBase (Froese & Pauly, 2013). Habitat is the predominant type recorded in FishBase. Constants for the regression equation fitted to the species and depth data are given in the last three columns

				Depth (m)				Log_10_ species regression		
	Order	N	Family	Spp.	Maximum	Mean	Habitat	Slope	*k*	*r*^2^
Lower Teleosts	Notacanthiformes	3	Notacanthidae	11	4560	2092	Demersal	−0·000094	0·488	0·501
	Notacanthiformes	4	Halosauridae	16	5029	2013	Pelagic	−0·000115	0·620	0·531
	Anguilliformes	7	Synaphobranchidae	38	5440	1798	Demersal	−0·000135	0·829	0·450
	Anguilliformes	10	Serrivomeridae	11	6000	2595	Pelagic	−0·000036	0·246	0·107
	Saccopharyngiformes	20	Monognathidae	15	5800	3274	Pelagic	0·000007	0·174	0·005
	Saccopharyngiformes	23	Saccopharyngidae	10	3000	1751	Pelagic	−0·000116	0·672	0·481
	Osmeriformes	32	Platytroctidae	39	5393	1776	Pelagic	−0·000185	0·987	0·423
	Osmeriformes	33	Microstomatidae	19	4145	1044	Pelagic	−0·000203	0·822	0·697
	Osmeriformes	36	Opisthoproctidae	19	4750	1424	Pelagic	−0·000087	0·466	0·170
	Osmeriformes	37	Bathylagidae	22	4000	1526	Pelagic	−0·000144	0·677	0·629
	Osmeriformes	39	Alepocephalidae	94	5850	2048	Demersal	−0·000136	1·047	0·234
	Stomiiformes	40	Gonostomatidae	31	5301	2692	Pelagic	−0·000027	0·440	0·033
	Stomiiformes	42	Stomiidae	285	5087	1290	Pelagic	−0·000303	1·939	0·867
	Aulopiformes	46	Notosudidae	17	3000	1161	Pelagic	−0·000067	0·483	0·043
	Aulopiformes	48	Scopelarchidae	18	4740	2116	Pelagic	0·000000	0·251	0·000
	Aulopiformes	57	Paralepididae	59	4750	1168	Pelagic	−0·000237	1·243	0·795
	Myctophiformes	63	Myctophidae	249	6000	1092	Pelagic	−0·000306	1·902	0·890
Paracanthopterygii	Gadiformes	74	Macrouridae	398	6945	1299	Demersal	−0·000335	2·176	0·929
	Ophidiiformes	83	Aphyonidae	23	5610	3741	Demersal	−0·000001	0·354	0·000
	Ophidiiformes	85	Ophidiidae	249	8370	1532	Demersal	−0·000208	1·721	0·830
	Lophiiformes	91	Linophrynidae	29	2250	1328	Pelagic	0·000181	0·221	0·154
	Lophiiformes	93	Oneirodidae	65	3600	1550	Pelagic	−0·000178	1·035	0·215
	Lophiiformes	98	Himantolophidae	21	3000	1257	Pelagic	−0·000146	0·469	0·413
	Lophiiformes	103	Gigantactinidae	23	5300	1796	Pelagic	−0·000086	0·473	0·163
	Lophiiformes	104	Chaunacidae	16	2200	1062	Demersal	−0·000109	0·573	0·125
Acanthopterygii	Stephanoberyciformes	121	Melamphaidae	61	5320	1870	Pelagic	−0·000196	1·089	0·559
	Cetomimiformes	132	Cetomimidae	30	6200	2219	Pelagic	−0·000100	0·558	0·258
	Zeiformes	133	Oreosomatidae	10	1900	1330	Pelagic	0·000301	0·050	0·750
	Scorpaeniformes	166	Liparidae	382	7703	1359	Demersal	−0·000283	2·127	0·929
	Perciformes	205	Chiasmodontidae	32	5691	2136	Pelagic	−0·000043	0·486	0·036
	Perciformes	211	Zoarcidae	288	5320	1243	Demersal	−0·000359	2·151	0·924

Spp., the number of species per family listed in FishBase.

The deepest species within the lower teleosts was the eel *Serrivomer brevidentatus* Roule & Bertin 1929 recorded from 6000 m. *Abyssobrotula galatheae* (Ophidiidae), the world's deepest fish at 8370 m, belongs to the Paracanthopterygii and the deepest acanthopterygian was the liparid, *P. amblystomopsis*, found at 7703 m (Fujii *et al*., 2010).

**Fig 6:**
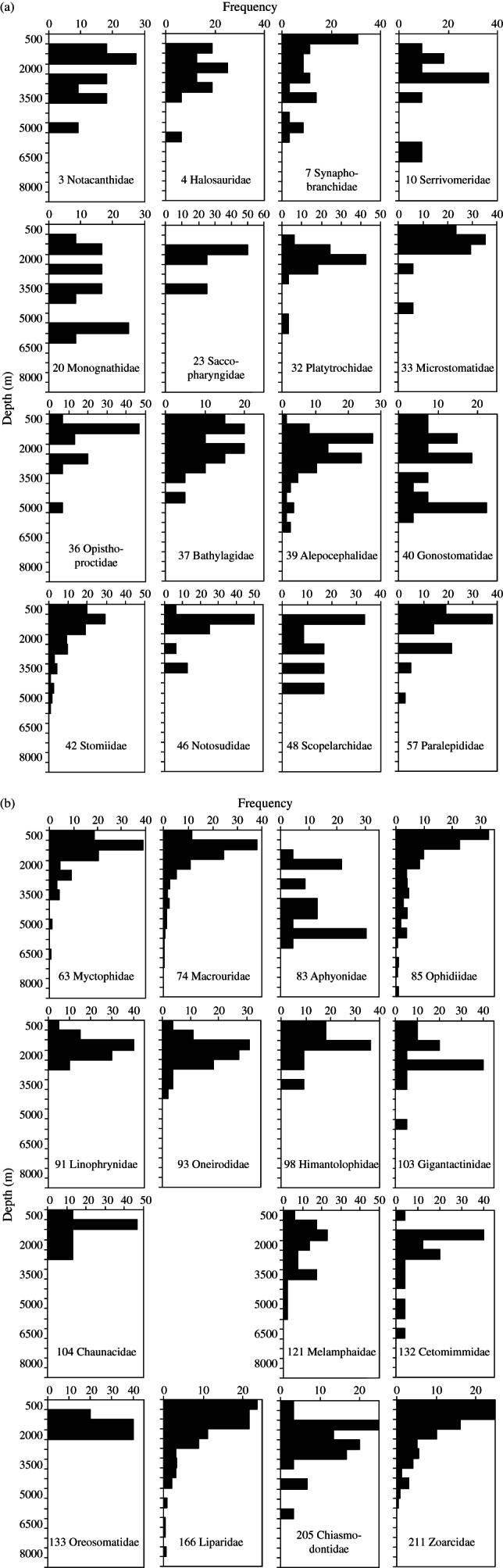
Depth-frequency distributions of fishes in the 31 deep-sea actinopterygian families ranked by order (N) given in Table2, *i.e*. families with a mean maximum depth of occurrence >1000 m and >10 species. (a) The lower families Notacanthidae to Paralepididae, (b) Myctophidae, Paracanthopterygii (Macrouridae to Chaunacidae) and the Acanthopterygii (Melamphaidae to Zoarcidae).

The depth frequency distributions of the 31 deep-sea families are shown in Fig. 6. Regression equations of the form log_10_*N* = *mx* + *k*, have been fitted to the distributions, where *N* is the number of species within the depth interval, *m* is the slope (rate of change in species number with depth), *k* is a constant (log_10_ number of species at zero depth) and *x* is depth (m). These values for each family are given in Table2. Families such as the Liparidae (166) with slope values <−0·0003 and *r*^2^ > 0·8 have maximum species numbers at shallow depths and a good fit to the general model of logarithmic decrease in species number with depth. These can be regarded as deep-sea invasive families. Families such as the alepocephalids (39) with a peak in numbers at depths below 1000 m show a poor fit to the model and have slope values that are positive (Linophrynidae, 91) or >−0·0001. These can be regarded as deep-sea endemic families that have either lost their presumed shallow-dwelling family members through extinction or have simply diverged so much from their shallow-water ancestors as to form a new family. Examining *r*^2^ and slope values in Table2, it is evident that all the three divisions of teleosts, lower, Paracanthopterygii and Acanthopterygii, contribute families across the spectrum between invasive and endemic. Correlations between family number and slope and *r*^2^ in Table2 are both insignificant (*r*^2^ = 0·0046 and 0·0259, respectively) indicating that taxonomic position is no predictor of whether a deep-sea family is endemic or invasive. For several of the families designated as invasive by this analysis, in Macrouridae, Stomiidae and Myctophidae, the peak of distribution of maximum depths lies at continental slope or mesopelagic depths of 500–1000 m rather than between the surface and 500 m.

## DISCUSSION

There is a clear global trend of logarithmic decrease in number of fish species with depth. The slope constant of the relationship between log_10_*n* and depth (−0·000422) predicts a 2·6 fold decrease in species per km increase in depth. This is very similar to estimates of rate of change in pelagic (Roe, 1988) and benthic biomass (Rex *et al*., 2006; Wei *et al*., 2010) with depth which show an overall mean slope of −0·00043 (Table3). Priede *et al*. (2013) show that pelagic and benthic biomass are linked to one another and are both determined by the availability of export food energy from the surface (Smith *et al*., 2009). With increasing distance from the primary producers in the euphotic zone, less biomass can be supported, fish populations become sparser and it appears that global fish biodiversity follows this depth-related energy constraint. Therefore, a primary hypothesis is proposed that the distribution of fish species with depth is governed by availability of energy.

**Table 3:** Trends of decrease in deep-sea pelagic and benthic biomass. List of slopes (*s*) in the general equation: log_10_*B* = *sx* + *k*, where *x* is the depth (m) and *k* is a constant

Biomass	Slope	Reference
Plankton and nekton (mean)	−0·00048	Roe (1988)
Benthic megafauna	−0·00039	Rex *et al*. (2006)
Benthic megafauna	−0·00031	Wei *et al*. (2010)
Benthic macrofauna	−0·00045	Rex *et al*. (2006)
Benthic macrofauna	−0·00052	Wei *et al*. (2010)
Mean	−0·00043	

The slopes of the regression lines for Myxini and Actinopterygii, 0·000488 and 0·000401 respectively, follow essentially the same energy-limited slope as observed in the entire global ichthyofauna. Chondrichthyes, however, show a much steeper slope of 0·000731, with species number decreasing by a factor of 5·4 for every 1000 m depth increment indicating that this class is in some way deficient in its ability to colonize the deep sea. Priede *et al*. (2006) suggested that Chondrichthyes might be limited by high energy requirements for buoyancy from an oil-rich liver. Such energy-limitation, however, would imply a slope closer to that for other classes of fishes. There may be other physiological limitations such as the need to use trimethylamine-N-oxide (TMAO) for maintaining osmotic balance and to confer pressure tolerance on vital proteins (Yancey & Siebenaller, 1999). Alternatively, there may be reproductive limitations or a combination of factors. Many deep-sea teleost species such as macrourids (Marshall, 1965) produce buoyant eggs so that planktivorous larvae develop in the surface layers of the ocean where copious food is available. Such a strategy is not available to Chondrichthyes owing to their large egg size and low fecundity. Planktotrophic development is only possible if inevitable high mortality is compensated for by high fecundity. Chondrichthyes invest all their reproductive effort in a few eggs with enough yolk to support direct development. Body size may also be a factor, mean maximum body *L*_T_ for the Chondrichthyes (108·5 cm) is much larger than for Actinopterygii (33·4 cm), and may be above the optimal size for survival at abyssal depths (Collins *et al*., 2005).

Comparing the three classes, the depth attained [Myxini (2743 m), Chondrichthyes (4156 m) and Actinopterygii (8370 m)] was correlated with the number of species 78, 1141 and 15 085 in the respective taxa. In all three classes, there is a maximum diversity at shallow depths suggesting that shallow seas act as centres of speciation. Habitat heterogeneity and intense competition in upper slope and shelf seas are likely to create possibilities for fractioning of populations and evolution of new species (Bowen *et al*., 2013), some of which may be relocated into the deep sea. The number of species moving into the deep sea would then be determined by the total number of shallow-water species and their speciation rate. A secondary hypothesis is proposed that the slope of the species–depth relationship may then be determined by the balance between colonization and extinction rates in the deep sea. Thus, the Actinopterygii reach the greatest depths in the ocean (Fig. 2) by virtue of their high species number and presumed high speciation rate. Theoretically, if Myxini were as numerous as the Actinopterygii they would attain the same depth. Chondrichthyes may be disadvantaged by an intrinsically low speciation rate or high extinction rate owing to physiological limitations. It is notable that in all three classes (Fig. 2) the length frequency distribution is log-normal (Froese, 2006) and that fishes at the greatest depths tend towards mean size.

**Fig 7:**
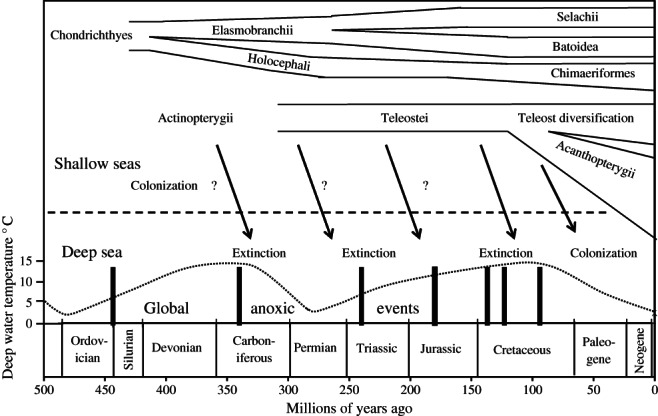
The geological time scale and evolution of fishes. 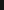 indicate global anoxic events (after Takashima *et al.*, 2006) when the deep sea is unlikely to have been habitable by fishes. Deep-sea bottom temperatures are indicated by the 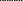 and scale on the left (Schopf, 1980). Times of divergence of the major classes, subclasses and divisions of fishes are taken from recent literature. 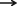 indicate the probable pattern of colonization of the deep from shallow seas but with localized and global extinctions in the deep. ? indicate putative pre-Cretaceous colonisation for which there is no direct evidence.

The relationship between evolution of fishes and the history of the oceans is summarized in Fig. 7. The deep ocean is prone to stagnation, if no water in the upper 1000 m becomes sufficiently dense to displace deeper water then the stratification is stable and the deep waters tend to become anoxic (Schopf, 1980). A modern example is the Black Sea (Wilkin *et al*., 1997) which is anoxic and devoid of metazoan life beyond a few hundred m depth. Throughout the Earth's history, there has been a series of global ocean anoxic events, when it is presumed that all conventional deep-sea metazoan life must have been extinguished (Takashima *et al*., 2006). There is a general consensus that the modern deep-sea benthic invertebrate fauna has originated from shallow waters following elimination of ancient deep-sea faunas by mass extinction events during the Jurassic and Cretaceous anoxic periods (Rex & Etter, 2010). Nevertheless, Thuy *et al*. (2012) report discovery of fossil echinoderm assemblages from 114 mya at 1000–1500 m paleodepth in the north-west Atlantic Ocean with a modern composition indicating possible survival through the most recent Cretaceous global oceanic anoxic event known as OAE2, Cenomanian–Turonian (Yilmaz *et al*., 2010) *c*. 94–96 mya. Raup & Sepkoski (1986) estimate that this event resulted in extinction of 50% of marine genera and 15% of families; even mass extinction events do not result in total loss of fauna. There is no doubt that deep-sea families would be particularly vulnerable to OAEs. In addition to global anoxic events, regional anoxia occurs at a higher frequency. For example, on the floor of the eastern Mediterranean Sea, sapropel layers rich in organic matter indicate deep-water anoxic events at intervals of tens of thousands of years. During the Messinian salinity crisis 5·96 to 5·33 mya, the Mediterranean Sea basin dried out, reducing sea level by 1500 m creating salt deposits and anoxic brine pools (Garcia-Castellanos & Villasen, 2011) totally eliminating the marine fauna until re-colonization when the sea re-filled (Garcia-Castellanos *et al*., 2009). An alternative to the stagnant ocean basin model is expansion of oxygen minimum layers creating regional anoxia in an otherwise oxic ocean above and below (Takashima *et al*., 2006). Such events have occurred in the eastern Pacific Ocean during the Miocene (10–20 mya) forming barriers to fish movement that have been implicated in speciation in the deep sea (White, 1987). Thus, whilst the deep sea is often perceived as a stable environment, on a geological time scale it has been a transitory habitat.

Further changes have occurred associated with the process of break-up of the supercontinent Pangaea from *c*. 200 mya and the first opening up of the Atlantic Ocean *c*. 150 mya (Sheridan *et al*., 1982). Initially, the Atlantic Ocean consisted of separate northern and southern enclosed basins. Deep-water connections between the north and south Atlantic Ocean did not appear until 80 to 65 mya and essentially modern deep-water circulation was not established until 35 mya (Schopf, 1980). The depth of the ocean floor has also increased. During sea floor spreading, new oceanic crust is formed at a depth of *c*. 2600 m and as the hot basalt cools it contracts, increasing the ocean depth to over 5500 m after 70 million years. The Atlantic Ocean attained its current bathymetry *c*. 10 mya. Beyond 6000 m, the hadal zone is confined to oceanic trenches and troughs, the total area of which is <1% of the sea floor. A total of 37 hadal trenches and troughs have been identified, five in the Atlantic Ocean, four in the Indian Ocean and 28 around the Pacific Ocean rim (Jamieson *et al*., 2010). Trench formation is dependent on movement of tectonic plates; so this has been contemporary with movement of the continents into their current positions.

A characteristic feature of the modern deep-sea is that bottom water temperatures are low (typically 0–4° C); however, throughout earth history, there have been considerable fluctuations in bottom water temperature. Whilst surface temperature in tropical regions has been remarkably constant, 20–25° C, for over 700 million years, tropical bottom water temperature has oscillated between 15° C or more during warm periods in the Cambrian (500–600 mya), Devonian-Carboniferous (350 mya) and Cretaceous (100 mya), and 2° C during cold glacial periods in the Ordovician (450 mya), Permian (270 mya) and the present day (Schopf, 1980) (Fig. 7). In the North Atlantic Ocean, it is estimated that bottom water temperatures may have reached 20° C during the Cretaceous (Huber *et al*., 2002). Since that time, global deep waters have cooled to present-day values. Thus, during the initial period of colonization of the deep sea by modern fish taxa in the late Cretaceous, there would have been a small temperature gradient to overcome between the surface and the deep-sea floor. Deep water in the Mediterranean Sea and Red Sea remains at a temperature of 13–15° C as circulation is maintained by evaporation and sinking of warm saline water in contrast to oceanic deep-water formation by cooling (Schopf, 1980).

The major fish taxa originated long before the post-Cretaceous colonization of the deep sea, so their history in relation to palaeoceanography needs to be considered to understand present-day distributions. The origins of the hagfishes, Myxini, have been much debated Janvier (2010). It is now accepted that they are related to lampreys and other vertebrates but have undergone a process of degeneration from an anatomically more complex common ancestor (Heimberg *et al*., 2010). Fossil myxinids have been found in the late Carboniferous (Bardack, 1991) with a body form very similar to living species, indicating little change for over 300 million years and absence of new diversification. It is not possible to recognize deep-water specialists morphologically in the group, because all myxinids are anatomically very similar. The ability of myxinids to tolerate severe anoxia (Hansen & Sidell, 1983; Cox *et al*., 2011) suggests that they may have occupied the deep-sea ocean margins throughout their history with little evolutionary change in the face of advance and regression of deep-water oxygenation.

Molecular clock data (Licht *et al*., 2012) indicate that Holocephali diverged from the Elasmobranchs just over 400 mya near the beginning of the Devonian period, the so-called Age of Fishes. The Holocephali then diversified during the Jurassic, the Chimaeridae appearing *c*. 100 mya in the Cretaceous. For the modern Holocephali, the peak of distribution (Fig. 3) is between 500 and 1500 m depth suggesting this is a true deep-sea group of fishes. Molecular clock estimates show important speciation events after the last Cretaceous global OAE2: *Rhinochimaera* and *Harriotta* branch at 58 mya, *Hydrolagus* and *Chimaera* at 25–81 mya and *Callorhincus* species at 8–11 mya. It appears that whilst deep-sea Holocephali may be a truly ancient group there has been a considerable recent diversification. Studies on the physiology of the spotted ratfish *Hydrolagus colliei* (Lay & Bennett 1839) (Speers-Roesch *et al*., 2006) show that the energy metabolism pathways are very similar to those of elasmobranchs. Speers-Roesch & Treberg (2010) suggest that Holocephali and elasmobranchs have retained the metabolic organization that was present in the common ancestor of the Chondrichthyes over 400 mya. Despite their deeper distribution compared with other Chondrichthyes (Fig. 3), there is no evidence of distinctive physiological adaptations in the Holocephali.

The Selachii and Batoidea diverged from one another during the late Permian *c*. 260 mya. Modern orders of sharks and rays radiate from the Jurassic (160 mya) onwards to produce the present-day species, with >50% of species living at <500 m depth (Fig. 3). In the Selachii and Batoidea, the species-depth distribution fits the concept of recent colonization of the deep sea by species export from shallow seas.

The earliest actinopterygian fossil has been found in the mid-Devonian *c*. 380 mya and molecular clock data indicate that Teleostei appeared during the Carboniferous to early Permian, 333 to 285 mya (Near *et al*., 2012). This estimate is much earlier than the oldest known teleost fossils, elopomorphs and ostarophysians of the late Jurassic *c*. 180 mya. A major difficulty impeding interpretation of the early history of the Actinopterygii is the ‘teleost gap’, a lack of fossils from the mid-Carboniferous to early Triassic (Hurley *et al*., 2007; Near *et al*., 2012). The late Jurassic and early Cretaceous mark the onset of major diversification of the teleosts; the Acanthopterygii diverging *c*. 130 mya and many teleost taxa appearing during the Paleogene, which Near *et al*. (2012) propose should be known as the ‘Second Age of Fishes’. It is hypothesized that genome duplication triggered the origin of teleosts and their subsequent radiation. Santini *et al*. (2009) also detected an additional series of pulses of radiation notably 65 to 23 mya. They caution against ascribing these recent changes entirely to genetic mechanisms, pointing out that major environment changes, such as establishment of scleractinian coral reefs in tropical shallow habitats, continental drift, sea level fluctuations and oceanographic changes, all probably have had an effect. The time period since the Cretaceous OAE2, 94 mya, therefore is marked by cooling, reoxygenation and deepening of the abyssal ocean basins coinciding with an explosion of teleost diversification, particularly the Acanthopterygii, including some colonization of the deep sea. Myxini, Holocephali, early Elasmobranchs and Actinopterygii may have inhabited ancient deep seas from the end of the Devonian onwards when conditions were suitable, but continuous occupation through mass extinctions seems unlikely. All groups except the Myxini show important recent diversification.

Within the Actinopterygii, there is a trend that more primitive orders (in the terminology of Andriashev, 1955) are more likely to have deep-sea representatives (Figs 4 and 5). Passage of time has presumably given more opportunities to colonize the deep sea but absence of a thermocline in the Cretaceous ocean and lack of competition in the deep sea after the OAE2 extinctions may have been more conducive to deep-sea colonization than in the modern ocean. Interpretations, however, are affected by taxonomy. This study has followed the phylogenetic sequence in FishBase (Froese & Pauly, 2013) and has used the Paracanthopterygii as a convenient intermediate group between the ancient orders and the Acanthopterygii. It is doubtful, however, if it is a monophyletic (Nelson, 2006) and molecular phylogeny now breaks up the Paracanthopterygii placing Lophiiformes with the Tetraodontiformes amongst the most recent of Acanthopterygii (Near *et al*., 2012). Ophidiiformes and Batrachiformes remain together, but the Gadiformes and Zeiformes become sister groups just below the Myctophiformes (Table1). Following this revision, the Lophiiformes with quintessential highly specialized deep-sea species such as angler fishes of the superfamily Ceratioidea with their dwarf males are placed as very recent arrivals in the deep sea, contrary to the ideas of Andriashev (1955). Molecular clock data indicate divergence of the Lophiiformes at the end of the Cretaceous (66 mya) and Carnevale & Pietsch (2009) describe a fossil linophrynid from Miocene chalk deposits of southern California (10–20 mya). These revisions of positions of different orders are not sufficient to disrupt the general conclusions from Figs 3 and 4 that older orders have a larger proportion of species in the deep sea, but fish phyologeny is currently in a state of flux (Nelson, 2006). The Miocene beds of southern California harbour a rich assemblage of oceanic and deep-sea fishes including caristiids, lampridids, melamphaids, sternoptychids, myctophids and stomiids (Carnevale & Pietsch, 2009) indicating that by this time many features of the modern deep-sea ichthyofauna had become well established, contemporary with appearance of the first apes on land.

In this study, analysis of deep-sea families (Fig. 6) identified two types of depth-frequency distribution, invasive and deep-sea endemic. The invasive families (*e.g*. Synaphobranchidae, Ophidiidae and Liparidae) have most of their representatives in shallow seas with a tail of the distribution extending into the deep sea, whereas the deep-sea endemic families are those that have their centre of distribution at depths >1000 m and have few or no species living at shallow depths (*e.g*. Serrivomidae, Monognathidae, Alepocephalidae, Aphyonidae and Chiasmodontidae). It is hypothesized that in the invasive families, colonization from shallower waters replaces species that become extinct in the deep sea. Thus, the persistence of the taxon in the deep sea is by virtue of constant replenishment from above. Although identified as invasive families, the Myctophidae, Macrouridae and Zoarcidae have a deeper peak of depth distribution between 500 and 1000 m. The centre of speciation may be on the upper slope or in the mesopelagic with species exported upwards into shallow water as well as down towards the abyss.

It is paradoxical that the invasive families with most species in shallow seas are also the main contributors to the hadal ichthyofauna at depths >6000 m (Fujii *et al*., 2010). The hadal fauna is characterized by the presence of regionally endemic liparids. Thus, in the north-west Pacific Ocean, the Japan and Izu-Bonin trenches are populated by *P. amblystomopsis* (Andriashev, 1955), whereas south-west Pacific Kermadec and Tonga Trenches are populated by *N. kermadecensis*. It is likely that these are the results of independent speciation events. The four species of the genus *Notoliparis* are endemic to four different trench systems in the southern hemisphere indicating allopatric speciation in the deep sea and colonization by distinctive hadal forms from trench to trench. The hadal trenches and basins may be regarded as an archipelago of discrete habitats colonized in accordance with equilibrium theory of island biogeography (MacArthur & Wilson, 1963). The Ophidiid, *A. galatheae*, generally recognized as the world's deepest fish, is the extreme representative of an invasive family with >30% of species shallower than 500 m depth but with a significant tail of its depth distribution extending into the deep sea [Fig. 6(b)]. The Ophidiidae are a conspicuous component of the abyssal demersal ichthyofauna and it is not surprising that they are represented at hadal depths beyond 6000 m. In contrast to hadal liparids, which have been repeatedly seen from manned and unmanned submersibles and captured in traps (Fujii *et al*., 2010), there has been no further confirmation of hadal occurrence of *A. galatheae* since capture of the first specimen by trawl. As this is a demersal species, capture during ascent of the net through the water column appears unlikely, so the original record (Nielsen, 1977) cannot be rejected lightly. Whatever the true status of *A. galatheae*, the maximum depth limit for fishes appears to be *c*. 8000 m. Reported observation of flatfishes at the bottom of the Marianas Trench at *c*. 10 900 m depth during the dive of the bathyscaphe *Trieste* in 1960 is now regarded as erroneous (Jamieson & Yancey, 2012). There may be ultimate physiological limits to the depth attainable by fishes but also the environment of the trench beyond 8000 m depth is dominated by increasing numbers of lysianassoid amphipods that swarm around artificial food falls (Blankenship & Levin, 2007; Jamieson *et al*., 2009) possibly competitively excluding fishes or indeed preying upon them.

The deep-sea endemic families generally have highly modified body forms specialized to life in the deep sea. Many species are global in their distribution and in this way can recolonize in the event of regional extinction events in contrast to the invasive hadal liparids which must be vulnerable to extinction by events within particular trench systems. Furthermore, Howes (1991) points out that within the gadoids circumglobal distribution is characteristic of the more primitive taxa and the phylogenetic trend has been a process of colonization from oceanic to shelf regions; so the derived taxa occupy shallow seas, which is actually the reverse of the generally accepted trend. A remarkable example of potential for oceanic to shallow-water diversification is in the eels. Inoue *et al*. (2010) show that the freshwater eels *Anguilla* spp. are most closely related to mesopelagic eels of the families *Nemichthyidae* and *Serrivomidae* (deep-sea endemics in the classification of this study) that spawn in the open ocean. *Anguilla japonica* Temminck & Schlegel 1846 has been found to spawn at 220–230 m depth in the western North Pacific Ocean, a trait apparently retained from their oceanic pelagic ancestry (Chow *et al*., 2009).

Within the teleosts, analysis in this study confirmed the general proposition of Andriashev (1955) with a greater depth of occurrence in the more ancient taxa (Figs 4 and 5). The dichotomy between the ancient (specialized) and secondary (non-specialised) deep-water fishes, however, is becoming blurred by advances in taxonomy. The Acanthopterygii are dominated by a vast diversity of shallow-water species but nevertheless contribute significantly to the deep-sea ichthyofauna including several deep-sea endemic families [Fig. 6(b)]. Whilst the Holocephali can be regarded as ancient deep-water forms, there seems to be no reason to separate the Selachii as ancient and the Batoidea as secondary; they appear to have colonized the deep sea synchronously.

A problem that remains here is the analysis that is based on data for maximum depth of occurrence which are prone to error, particularly arising from uncertainty of depth of capture where the fishing gear has traversed a large bathymetric range. Camera vehicles equipped with depth sensors provide unambiguous evidence of the depth of living (Jamieson *et al*., 2009), although species identification must be confirmed by capture of specimens. A high threshold (1000 m) was also used to define deep-sea families. This has excluded some families which make an important contribution to the deep-sea ichthyofauna, such as the Moridae (mean maximum depth of 808 m) and Trachichthyidae (mean maximum depth of 602 m), which include important commercial species such as the blue antimora *Antimora rostrata* (Günther 1878) and orange roughy *Hoplostethus atlanticus* Collett 1889 respectively. In this study, the 500 m depth increments fail to resolve the detail of the mesopelagic and upper slope ichthyofaunas which are often zones of high biodiversity (Priede *et al*., 2010); this was outside the scope of the present investigation.

Whilst there is good evidence of colonization of the deep sea from the Cretaceous onwards, there is no direct evidence of deep-sea fish faunas during periods of potential colonization between anoxic events from the Devonian to Jurassic (Fig. 7). For invertebrates, Thuy *et al*. (2012) found fossils indicating localized survival of deep-sea echinoderms through the Cretaceous OAE2 leading them to propose the possibility of a more ancient origin for deep-sea fauna. Continuous survival of deep-sea lineages through multiple anoxic events seems unlikely for fishes whose data are consistent with continuing colonizations from shallow seas over the past 100 million years. The question of a pre-Cretaceous deep-sea ichthyofauna remains an enigma.
